# KLF3 promotes colorectal cancer growth by activating WNT1

**DOI:** 10.18632/aging.205494

**Published:** 2024-02-01

**Authors:** Wei Shen, Lebin Yuan, Boyu Hao, Jiajia Xiang, Fei Cheng, Zhao Wu, Xiaodong Li

**Affiliations:** 1 Department of General Surgery, The Second Affiliated Hospital of Nanchang University, Nanchang 330006, Jiangxi, China; 2 General Medicine, First Affiliated Hospital of Nanchang University, Nanchang 330006, Jiangxi, China; 3 Laboratory of Molecular Center, The Second Affiliated Hospital of Nanchang University, Nanchang 330006, Jiangxi, China

**Keywords:** colorectal cancer, KLF3, WNT1, proliferation, invasion

## Abstract

Objective: The function of Kruppel-like factor 3 (KLF3) remains largely unexplored in colorectal cancer (CRC).

Methods: KLF3 expression in CRC was assessed through qPCR, western blotting, immunohistochemical assays, and The Cancer Genome Atlas (TCGA) database. The tumor-promoting capacity of KLF3 was explored by performing *in vitro* functional experiments using CRC cells. A subcutaneous nude mouse tumor assay was employed to evaluate tumor growth. To further elucidate the interaction between KLF3 and other factors, luciferase reporter assay, agarose gel electrophoresis, and ChIP analysis were performed.

Results: KLF3 was downregulated in CRC tissue and cells. Silencing of KLF3 increased the potential of CRC cells for proliferation, migration, and invasion, while its activation decreased these processes. Downregulated KLF3 was associated with accelerated tumor growth *in vivo*. Mechanistically, KLF3 was discovered to target the promoter sequence of WNT1. Consequently, the diminished expression of KLF3 led to the buildup of WNT1 and the WNT/β-catenin pathway activation, consequently stimulating the progression of CRC.

Conclusions: This investigation suggests that the involvement of KLF3/WNT1 regulatory pathway contributes to the progression of CRC, thereby emphasizing its promise as an important focus for future therapies aimed at treating CRC.

## INTRODUCTION

According to statistical data, CRC is globally ranked as the third most prevalent form of cancer and accounts for the second highest mortality rate among all types of cancers [1]. Globally, there are nearly 90,000 new cases of CRC, of which more than 9.1 million succumb to this disease [2]. Despite endless efforts to improve early detection and treatment approaches, the outlook for individuals with CRC remains less than satisfactory. Approximately 20% of CRC patients develop metastasis or recurrence [3]; hence, the discovery of novel biomarkers may make some critical advancements in diagnosing early cancer, pave new directions for the treatment of CRC, and provide ideas for the development of new targeted reagents.

Krüppel-like factor 3 (KLF3 or BKLF), initially discovered in erythroid cells, primarily governs gene expression through its interaction with the GC-rich promoter sequence known as the GC/GT box [4, 5]. KLF controls several essential physiological processes, such as angiogenesis, B lymphopoiesis, adipogenesis, and so on [6–8]. The occurrence and progression of various types of tumors, such as cervical cancer, have been linked to the atypical expression patterns exhibited by members of the KLFs family [9], pancreatic cancer [10], and stomach cancer [11]. KLF3 silencing, mediated through DNA methylation, has been observed to enhance metastasis in human sarcoma cells [12]. Increased accumulation of KLF3 through regulation of the miR-326/Sp1/KLF3 axis leads to the JAK1/STAT3 and PI2K/AKT signaling pathways activation, thereby facilitating the progression of lung cancer [13]. KLF3 was also seen as an oncogenic transcription factor in CRC in a genome-wide analysis of 73 CRC specimens [14]. Fan et al. analyzed the TCGA database and found suppression of KLF3 in CRC specimens and proposed its potential utility as an independent prognostic factor [15]. However, the precise mechanistic function of KLF3 in CRC has yet to be clarified.

According to these study findings, it is postulated that KLF3 plays a vital part in the promotion of CRC. This hypothesis, which correlated with a poorer prognosis, was substantiated by the observation of reduced levels of KLF3 in patients with CRC, determined through the analysis of patient tissue and CRC cells. Subsequently, the KLF3 function in the progression of CRC was elucidated by evaluating cellular proliferation, migration, and invasion. KLF3 was observed to bind directly to the WNT1 promoter, thereby inducing the WNT/β-catenin pathway and consequently contributing to the facilitation of CRC tumorigenesis and invasiveness.

## RESULTS

### Krüppel-like factor 3 expression is down-regulated in colorectal cancer and is related to unfavorable outcomes in such patients

**Figure 1:**
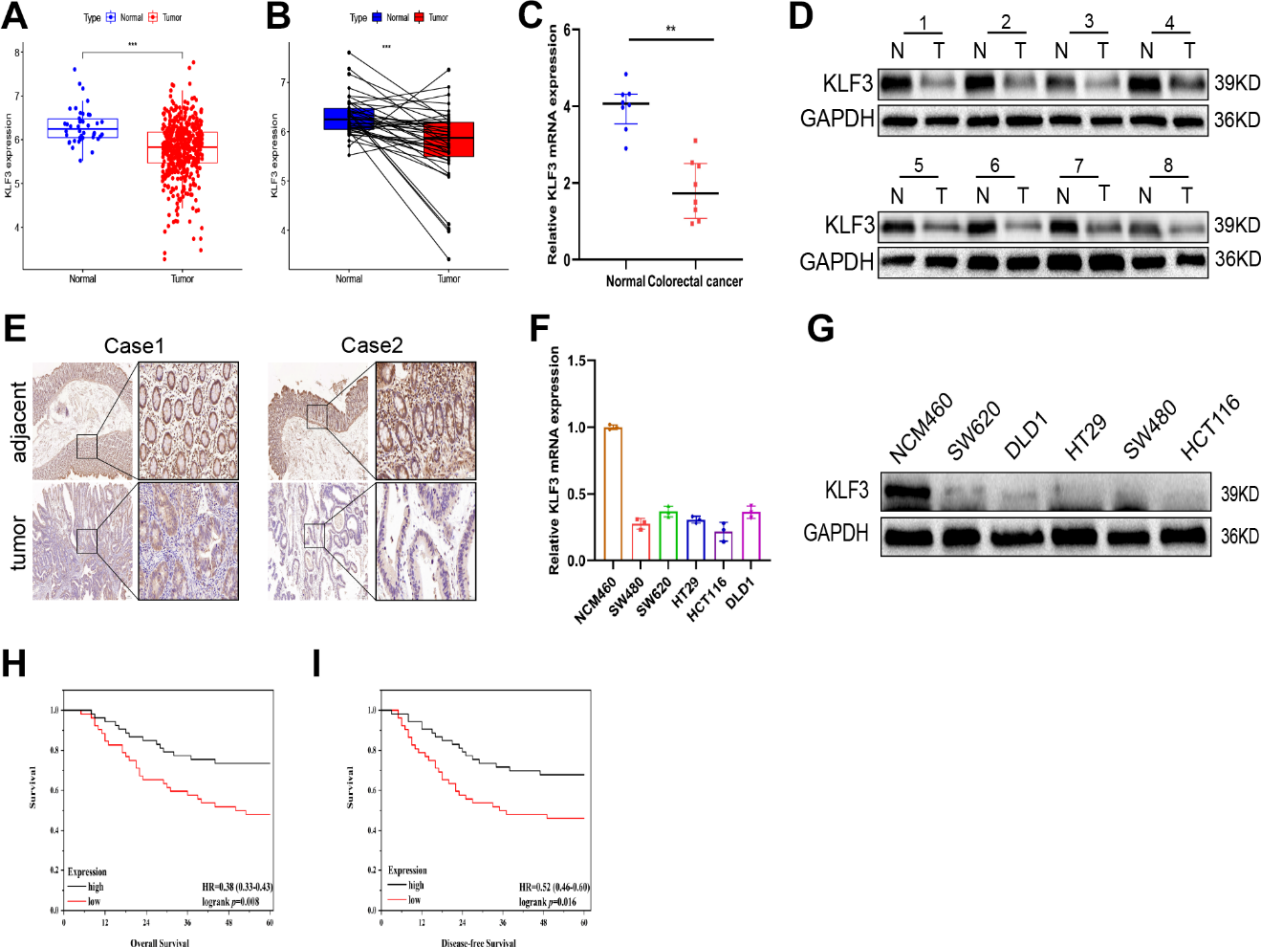
**Association of KLF3 down-regulation with prognosis in CRC patients.** (**A**, **B**) KLF3 expression was significantly lower in 488 CRC specimens compared to 42 adjacent non-CRC specimens, and in matched CRC specimens compared to matched normal colon specimens. The data is presented as (-log10) for KLF3 expression (fpkm). The line shows the average value. (**C**, **D**) mRNA and protein levels of KLF3 measured by qPCR and western blotting, respectively, in eight pairs of randomly selected CRC tissues and normal samples. (**E**) Immunohistochemistry analysis of the expression of KLF3 protein in CRC and normal samples. (**F**, **G**) KLF3 mRNA and protein expressions were higher in NCM460 than in normal CRC cell lines. (**H**, **I**) Kaplan-Meier curves showing reduced overall survival (OS) and disease-free survival (DFS) in patients with low KLF3 mRNA levels. Scale bars: 50 μm. *p < 0.05; **p < 0.01; ***p < 0.001.

We utilized RNA-seq data from TCGA to assess KLF3 expression in 488 CRC samples and 42 peritumor samples to investigate the potential function of KLF3 in tumorigenesis. As shown in Figure 1A, KLF3 expression was reduced in TCGA-CRC samples compared to control, and in paired CRC samples contrasted to the corresponding normal cohort (Figure 1B, p<0.05). Tissue samples (n=8) along with paired tumor-adjacent normal tissues were assessed through qRT-PCR and western blotting to analyze KLF3 expression. The results revealed a notable decrease in KLF3 expression in CRC tissues contrasted to its levels in neighboring normal tissues (Figure 1C, 1D and Supplementary Figure 1A p<0.05). Immunohistochemistry (IHC) further confirmed decreased levels of KLF3 protein in CRC tissues (Figure 1E). Further analysis of several CRC cell lines showed a significant reduction in KLF3 mRNA and protein expression contrasted to control, particularly in HCT116 and SW480 cells (Figure 1F, 1G and Supplementary Figure 1B, p < 0.01). Correlation analysis of a cohort of 105 patients implied a positive association between KLF3 expression and CEA level (p=0.041), TNM stage (p=0.015) and vessel/ nerve invasion (p=0.002, Table 1). The Kaplan-Meier curves demonstrated that patients who have higher levels of KLF3 mRNA exhibited more favorable overall survival (OS) and disease-free survival (DFS) in contrast to individuals with lower levels. This highlights the noteworthy prognostic significance of KLF3 (Figure 1H, 1I, p<0.001). These findings indicate that KLF3 is notably under-expressed in CRC samples, which correlates with an adverse prognosis for patients with CRC.

**Table 1:** Correlation between KLF3 levels in 105 CRC patients and their clinicopathological characteristics.

**Variables**	**Clinicopathological characteristics**	**Numbers**	**Low expression**	**High expression**	**p-value**
Age	≤60	49	22	27	0.375
	>60	56	30	26	
Gender	Female	57	30	27	0.488
	Male	48	22	26	
CEA(U/L)	≤5	50	30	20	**0.041**
	>5	55	22	33	
TNM stage	I	23	5	18	**0.015**
	II	33	20	13	
	III	34	17	17	
	IV	15	10	5	
Vessel/ Nerve invasion	Negative	47	31	16	**0.002**
	Positive	58	21	37	
Tumor location	Right half colon	45	26	19	0.500
	Left half colon	15	7	8	
	Sigmoid	18	7	11	
	Rectum	27	12	15	
Tumor size(cm)	≤5	70	30	40	0.053
	>5	35	22	13	

### Aberrant KLF3 expression affects growth, migration, and invasiveness of colorectal cancer HCT116 and SW480 cells

**Figure 2:**
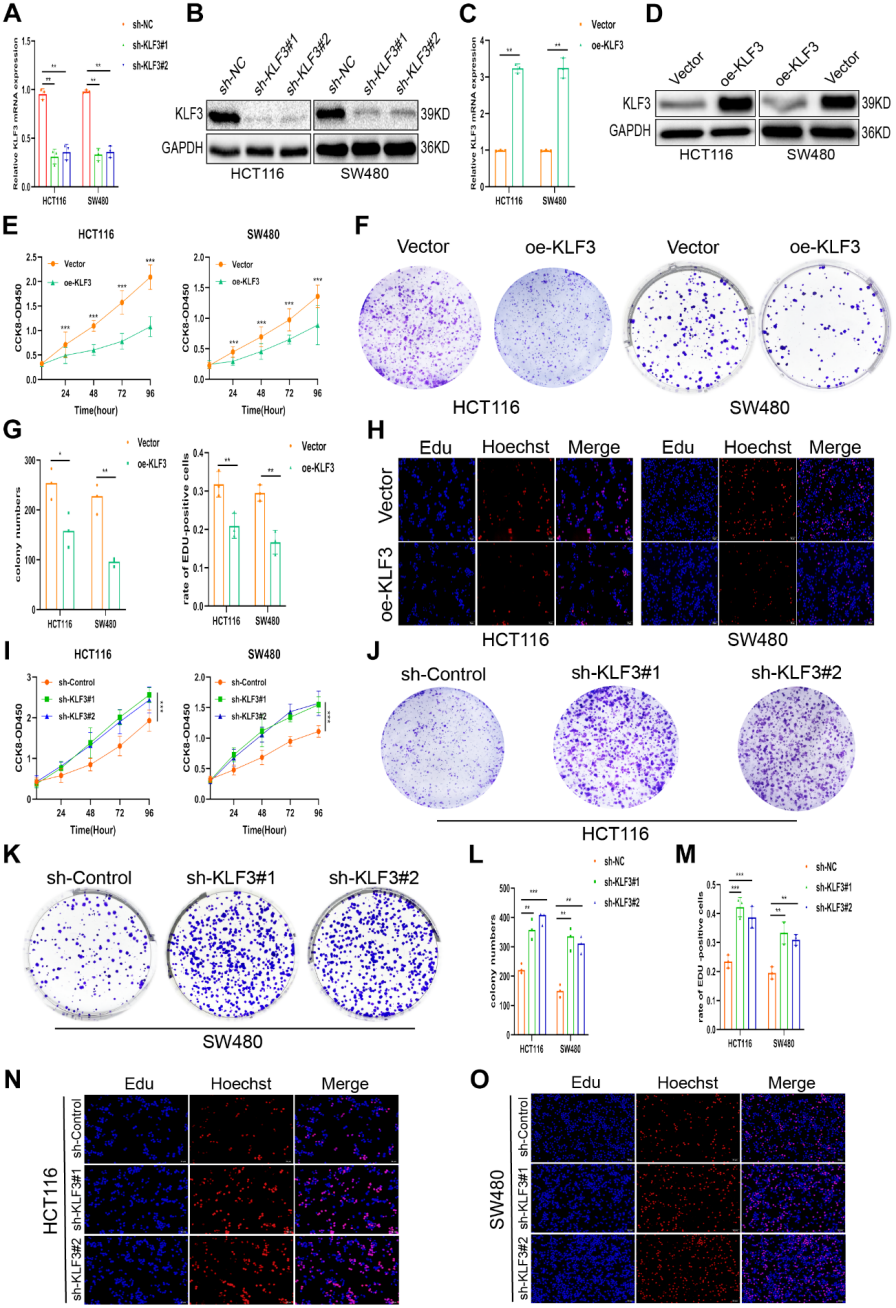
**KLF3 expression is associated with proliferation in CRC cells.** (**A**–**D**) The efficacy of knockdown and oe-KLF3 in HCT116 and SW480 CRC cells were evaluated. (**E**–**O**) Inhibition and promotion of cellular proliferation induced by KLF3 overexpression and knockdown, respectively, shown by CCK-8, EdU, and colony formation assays. Scale bars: 50 μm. *P<0.05, **P<0.01, ***P< 0.001.

**Figure 3:**
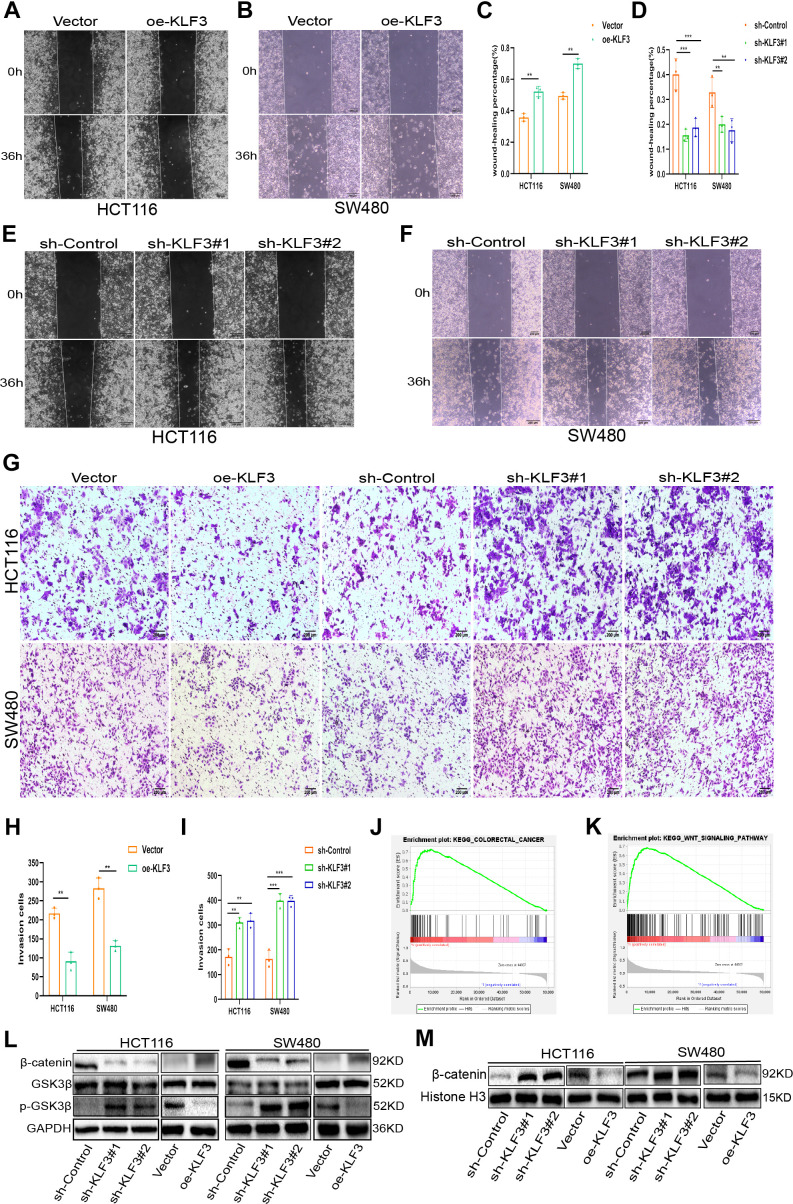
**KLF3 knockdown enhances invasion in CRC cells and activates the WNT/β-catenin axis.** (**A**–**F**) Representative wound-healing images showing migration in oe-KLF3 and KLF3-knockdown cells. The percentage was calculated according to the change in distance between each side. (**G**–**I**) Representative images showing the invasive capacity of CRC cells with up- and down-regulated KLF3 expression. (**J**, **K**) GSEA showing positive correlations between KLF3 expression, CRC progression, and the WNT/β-catenin axis. (**L**, **M**) Levels of markers associated with the WNT/β-catenin axis, including GSK3β, p-GSK3β (Ser9), and nuclear β-catenin in oe-KLF3 and KLF3-knockdown CRC cells. The data represent means ± standard deviation. Scale bars: 50 μm. **p < 0.01; ***p < 0.001.

Endogenous KLF3 expression was suppressed by a lentiviral vector carrying a specific shRNA against KLF3 in HCT116 and SW480 cells. Additionally, stable KLF3-overexpressing cell lines (oe-KLF3) of SW480/KLF3 and HCT116/KLF3, were established. Figure 2A–2D presents the results of qPCR and WB, demonstrating the excellent efficiency of KLF3 regulation, while the vector or control group had no such effect (Supplementary Figure 1C, 1D). Cell proliferation was monitored using CCK-8, colony-forming, and 5-ethynyl-2’-deoxyuridine (EdU) assays. Results indicated that oe-KLF3 significantly impaired the proliferation capacity of the cells (Figure 2E–2H). Conversely, reduced expression of KLF3 using small interfering RNA (sh-KLF3#1 and KLF3#2) increased proliferative capacity of both CRC cell lines (Figure 2I–2O). Subsequently, the influence of KLF3 on the invasion and migration abilities of CRC cells was explored. The obtained images and quantification information from the wound healing assay offered empirical evidence indicating that oe-KLF3 impeded the process of cell migration, while sh-KLF3 promoted migration in CRC cells (Figure 3A–3F). Transwell assay revealed that oe-KLF3 in HCT116 and SW480 cells led to reduced invasion ability; conversely, sh-KLF3 significantly enhanced cell invasion ability of both cancer cell lines (Figure 3G–3I).

### Aberrant expression of KLF3 alters the malignant behavior of colorectal cancer cells via the WNT/β-catenin axis

The precise mechanism associated with KLF3 was elucidated by performing GSEA analysis. The findings demonstrated notable enrichment of the CRC and WNT/β-catenin pathways in differentially expressing KLF3 (Figure 3J, 3K). Subsequently, Western Blot analysis was conducted to quantitatively evaluate the protein concentrations, including glycogen synthase kinase-3 beta (GSK3β), p-GSK3β(Ser9), and β-catenin, associated with the pathway in CRC cells. The altered levels of these proteins indicated that sh-KLF3 suppressed the expression of GSK3β, resulting in increased concentrations of p-GSK3β(Ser9) and nuclear β-catenin, and reduced cytoplasmic β-catenin levels in CRC cells. Conversely, in CRC cells harboring oe-KLF3, p-GSK3β(Ser9) and nuclear β-catenin levels were reduced, cytoplasmic β-catenin levels were increased, with relatively unaltered GSK3β expression (Figure 3L, 3M and Supplementary Figure 1E, 1F). The available evidence suggests that the WNT/β-catenin axis is affected by KLF3, which in turn impacts the development of CRC.

### Reduction of KLF3 promotes xenograft tumor growth in nude mice

**Figure 4:**
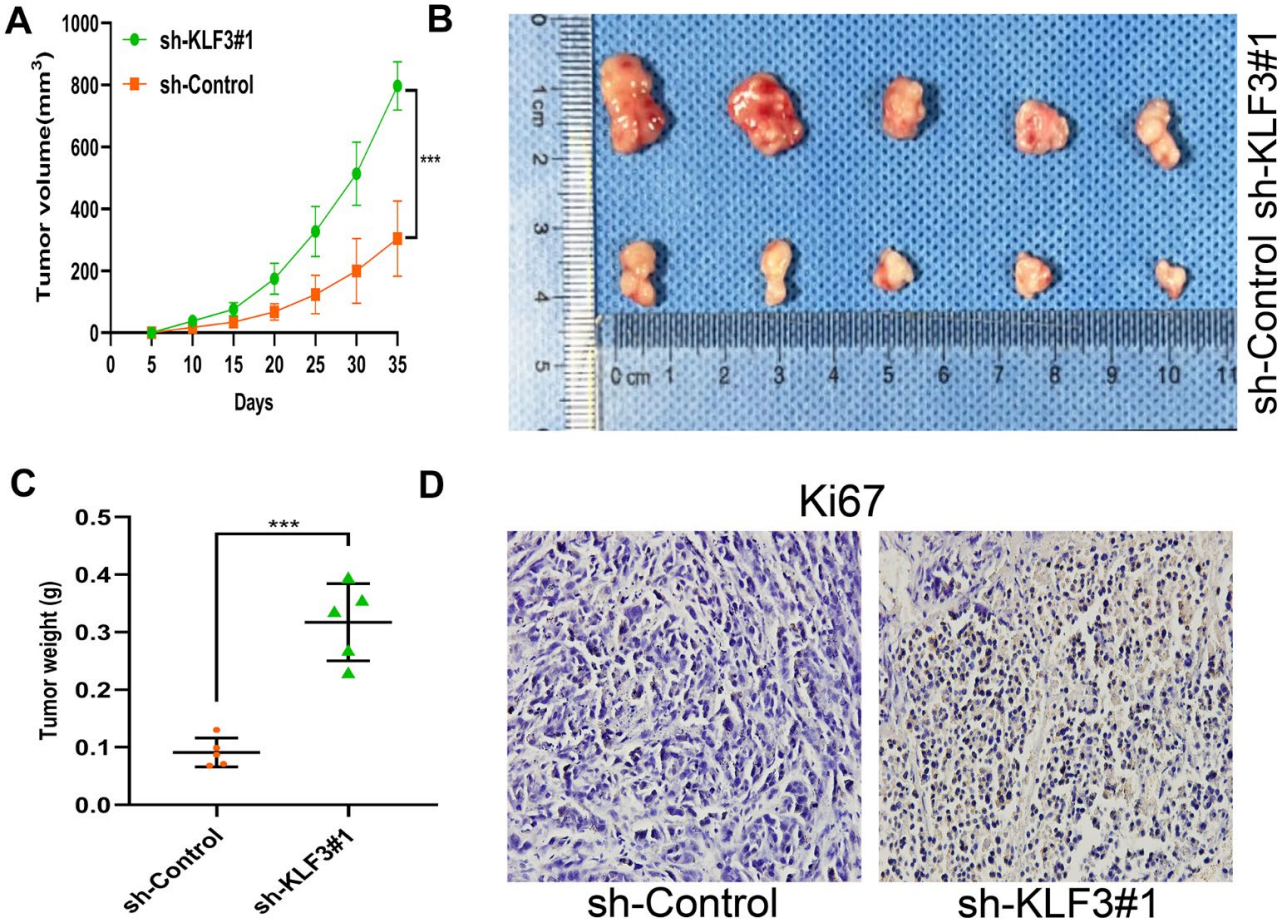
**Down-regulation of KLF3 promotes CRC growth *in vivo*.** (**A**–**C**) Representative images of HCT116-sh control or HCT116-sh-KLF3#1 subcutaneous xenograft tumor. Tumors in KLF3 knockdown mice grew faster than in controls in terms of volume and mass (N = 5/ group). (**D**) Ki67 level was elevated in HCT116-sh-KLF3#1 cell relative to HCT116-sh-NC cells. Scale bar: 20 μm. ***P< 0.001.

The effects of KLF3 on tumor development were examined by subcutaneously injecting BALB/c nude mice with HCT116 cells containing sh-KLF3#1. The suppression of KLF3 expression greatly increased tumor volume (Figure 4A, 4B, p< 0.05) and weight in a time-conditioned manner (Figure 4C, p< 0.05). The expression of Ki67, a proliferation marker, was examined in tumor specimens to assess the impact of sh-KLF3#1 on proliferation (Figure 4D). These results collectively demonstrate the oncogenic role of KLF3 in CRC progression through the improvement in proliferation, invasion, migration, and tumorigenesis of CRC cells.

### Negative correlation of KLF3 with WNT1 expression

**Figure 5:**
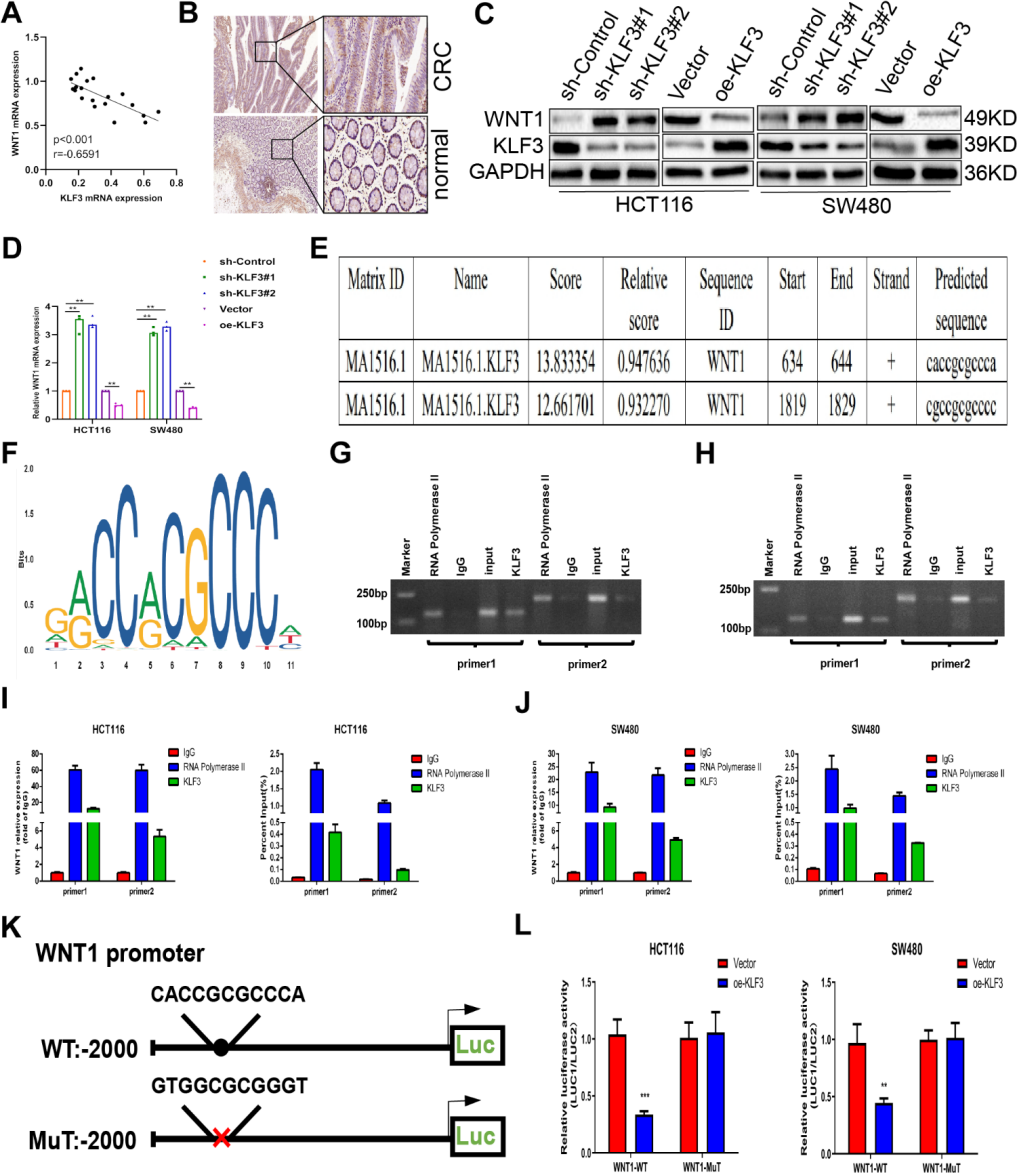
**Krüppel-like factor 3 (KLF3) correlates positively with WNT1 expression.** (**A**) Pearson’s correlations showing the relationship between KLF3 and WNT1 expression. (**B**) WNT1 levels were higher in CRC samples than in normal tissues, as shown by immunohistochemical analysis. (**C**, **D**) mRNA and protein levels of WNT1 and KLF3 in HCT-116 and SW480 CRC cell lines, shown by qPCR and western blotting, respectively, after KLF3 knockdown or overexpression. (**E**) Potential interaction sites between KLF3 and WNT1 according to the JASPAR database. (**F**) Diagram illustrating the predicted interaction site for KLF3 in the WNT1 promoter. (**G**–**J**) Verification of KLF3 interaction with the WNT1 promoter region in CRC cells, shown by ChIP-qPCR and agarose gel electrophoresis. (**K**, **L**) Luciferase assays exhibited a significant inhibition of luciferase activity the oe-KLF3 group within the wild-type WNT1 promoter relative to the controls. No response to oe-KLF3 was seen with the mutant WNT1 promoter. ****P< 0.0001.

Previous studies have revealed that KLF3 regulates CRC progression through the WNT/β-catenin pathway. Literature review and database analysis to further understand the mechanism underlying this regulation, revealed the role of WNT1 as a stimulator within the WNT/β-catenin signaling pathway [16, 17], while some investigations indicate that WNT1 accelerates the course of growth and metastasis in gastric carcinoma cell through transcriptional activation [11]. Moreover, other studies reported that elevated levels of miR-130a-3p led to a reduction in WNT1 expression, resulting in CRC growth inhibition and enhanced apoptosis [18, 19]. As shown in Figure 5A, analysis of the relationship between KLF3 and WNT1 expressions in 20 clinical samples, revealed a negative correlation between these two genes in CRC tissues (r=0.6591, p<0.001). IHC staining demonstrated enhanced levels of WNT1 in CRC compared to normal cells (Figure 5B). Based on these findings, we hypothesized that KLF3 possibly regulates WNT1 expression in CRC cells. Furthermore, the quantitative analysis indicated an increase in the WNT1 mRNA and protein levels in sh-KLF3 cells contrasted to the control group; conversely, oe-KLF3 caused a decrease of WNT1 levels in HCT116 and SW480 cells (Figure 5C, 5D, and Supplementary Figure 1G). Therefore, these results suggest a precise involvement of KLF3 and WNT1 in CRC.

### WNT1 is the direct downstream target of KLF3

The possible interaction between KLF3 and WNT1 was examined by means of the JASPAR database (http://jaspar.genereg.net/) to search for possible KLF3 binding sites in the WNT1 promoter region. As predicted, we could identify 14 potential binding sites (Table 2), and the top two strongest binding sites with a relative score > 0.8 (Figure 5E). These findings were validated by chromatin immunoprecipitation (ChIP), in which anti-KLF3 antibodies were used and subsequently followed by qPCR with primers for the WNT1 promoter region. ChIP and agarose gel electrophoresis analysis clearly indicated the interaction between KLF3 and the first promoter region of the WNT1 gene (Figure 5F–5J), supporting the hypothesis that KLF3 may regulate WNT1 expression by binding to its promoter region. Subsequently, we generated mutations in the specific location of the WNT1 promoter identified by KLF3 (Figure 5K), and luciferase reporter assay demonstrated that KLF3 overexpression did not exert any discernible effect on the luciferase activity of the WNT1 promoter with a mutation, while markedly impacting the luciferase activity of wild-type (WT) WNT1 promoter (Figure 5L). These findings suggest that WNT1, a direct downstream effector of KLF3, was regulated by KLF3 at the transcriptional level.

**Table 2:** Prediction of possible binding sites between KLF3 and the WNT1 promoter using the JASPAR website.

**Matrix ID**	**Name**	**Score**	**Relative score**	**Sequence ID**	**Start**	**End**	**Strand**	**Predicted sequence**
MA1516.1	MA1516.1.KLF3	13.833354	0.947636	WNT1	634	644	+	Caccgcgccca
MA1516.1	MA1516.1.KLF3	12.661701	0.932270	WNT1	1819	1829	+	Cgccgcgcccc
MA1516.1	MA1516.1.KLF3	6.83708	0.855880	WNT1	1905	1915	-	Gacccctcccc
MA1516.1	MA1516.1.KLF3	5.616034	0.839866	WNT1	1005	1015	--	Agccgagccta
MA1516.1	MA1516.1.KLF3	4.942289	0.831030	WNT1	1956	1966	-	Acccccgcccc
MA1516.1	MA1516.1.KLF3	4.591699	0.826432	WNT1	1779	1789	-	Ggcccagcccg
MA1516.1	MA1516.1.KLF3	4.287341	0.822441	WNT1	294	304	-	Tatcatgcccc
MA1516.1	MA1516.1.KLF3	4.246492	0.821905	WNT1	1590	1600	+	Actcacgcccc
MA1516.1	MA1516.1.KLF3	4.122957	0.820285	WNT1	313	323	+	Ggcagtgcccc
MA1516.1	MA1516.1.KLF3	3.389603	0.810667	WNT1	406	416	-	Gatcacgccat
MA1516.1	MA1516.1.KLF3	3.061778	0.806367	WNT1	1962	1972	-	Gggctcacccc
MA1516.1	MA1516.1.KLF3	3.000058	0.805558	WNT1	2038	2048	+	Gtctgcgcccc
MA1516.1	MA1516.1.KLF3	2.955157	0.804969	WNT1	2012	2022	+	Aaccacagccc
MA1516.1	MA1516.1.KLF3	2.880474	0.803990	WNT1	281	291	-	Caccacacaca

### KLF3 promotes a malignant phenotype in colorectal cancer cells by regulating WNT1

**Figure 6:**
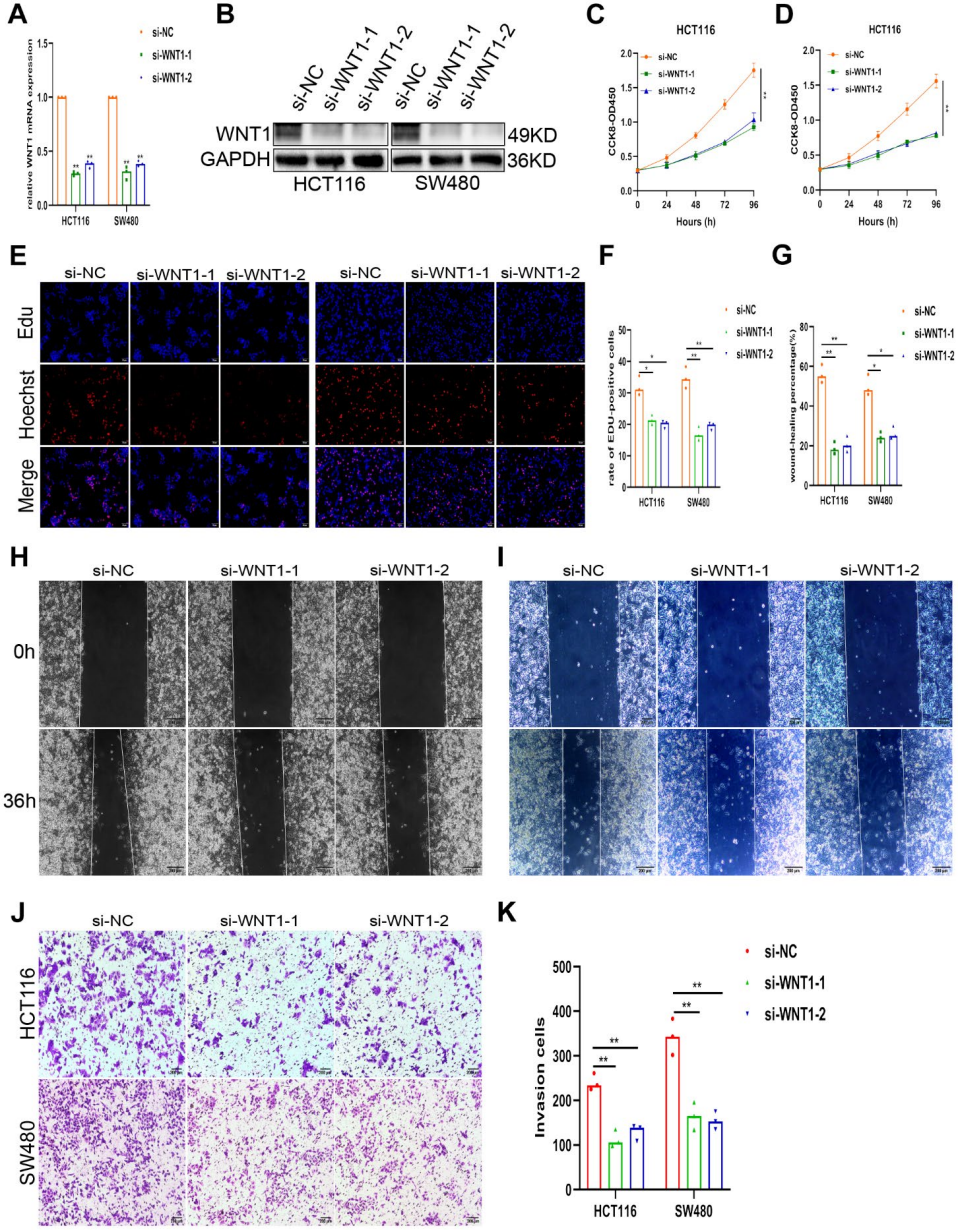
**Knockdown of WNT1 attenuates proliferation and invasion in CRC.** (**A**, **B**) WNT1 mRNA and protein levels in CRC cells following transfection with si-WNT1 or si-NC. (**C**–**K**) Alleviation of the malignant phenotype in CRC cells, shown by CCK-8, EdU, wound healing, and invasion assays. Knockdown of WNT reduced nausea in CRC cell lines HCT116 and SW480, as demonstrated by assays mentioned in (**C**–**K**). Scale bars: 50 μm, 200μm. *p < 0.05; **p < 0.01.

**Figure 7:**
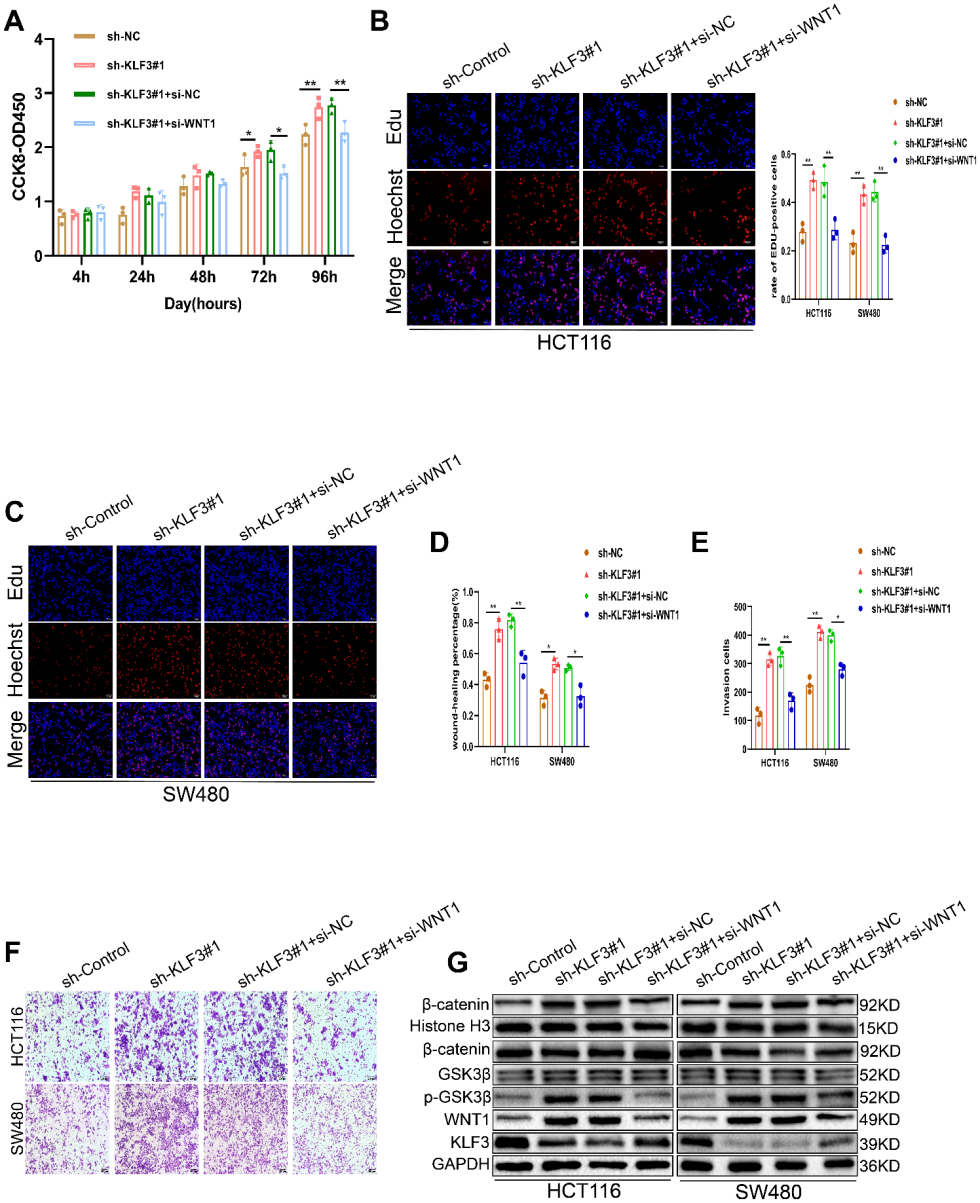
**WNT1 knockdown partially reverses the increased proliferation and invasion induced by sh-KLF3 transfection in CRC cells.** (**A**–**C**) CRC cells were transfected with sh-KLF3 prior to silencing using si-WNT1. Cell growth was measured by CCK-8 and EdU assays. (**D**–**F**) Cells were transfected with sh-KLF3 with or without si-WNT1 and assessed by wound healing and Transwell assays. (**G**) Levels of markers associated with the WNT/β-catenin axis, including GSK3β, p-GSK3β (Ser9), and β-catenin were reduced in KLF3-knockdown cells harboring si-WNT1. Data were analyzed by t-tests. Scale bars: 100 μm, 200 μm. *P<0.05, **P< 0.01.

To gain a deeper comprehension of the involvement of KLF3 in the regulation of WNT1-mediated growth of CRC cells, we utilized WNT1 siRNA and downregulated endogenous WNT1 expression in corresponding cells. WNT1 was efficiently knocked down, as shown in Figure 6A, 6B and Supplementary Figure 1H. Subsequent functional assessments demonstrated a significant decline in the proliferative, invasive, and metastatic capacities of CRC cells upon WNT1 knockdown (Figure 6C–6K). Next, investigation of the effects of WNT1 in KLF3-downregulated cells revealed that KLF3 downregulation increased the WNT1 expression, which in turn led to increased proliferation of CRC cells (Figure 7A–7C). Additionally, the observed reduction in migration and invasion in cells harboring sh-KLF3 was eliminated by suppressing WNT1 expression, as evidenced by the results of proliferation, wound healing, and invasion assay (Figure 7D–7F). Finally, we assessed the levels markers associated with WNT/β-catenin axis. KLF3 knockdown led to an enhanced expression of these markers, while WNT1 knockdown attenuated their expression (Figure 7G, Supplementary Figure 1I). KLF3 depletion upregulates WNT1 expression and activates WNT/β-catenin axis, thereby facilitating the proliferation, migration, and invasion of CRC cells.

## DISCUSSION

**Figure 8:**
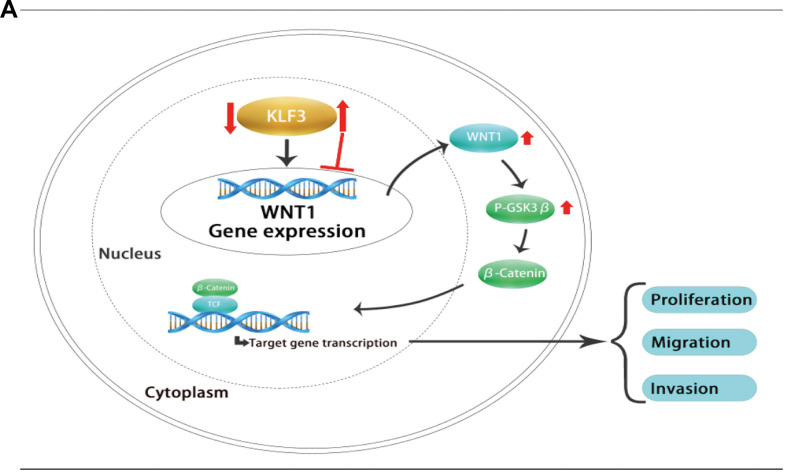
(**A**) Proposed mechanism by which KLF3 promotes CRC progression through activation of the WNT/beta-catenin axis by targeting WNT1.

The discovery of transcription factors was considered a hopeful approach in the field of cancer diagnosis and therapy. For example, breast and prostate cancer patients have benefitted greatly from studies on estrogen and androgen receptors [20, 21]. Targeted inhibitors such as Am80 have been developed to disrupt the KLF5-RARa interaction and inhibit KLF5-dependent transcription. Additionally, small-molecule inhibitors, such as PubChem compound ID 5951923, have been shown to decrease endogenous expression of KLF5 and cause a reduction in the proliferation in CRC cells [22, 23]. Here, our *in vitro* experiments confirmed that enhanced expression of KLF3 restrains the courses of CRC cell proliferation and invasion, consistent with previous findings [15]. We identified the prospective molecular mechanism by which KLF3 exerts its protective regulatory function in CRC progression, and it involves the modulation of WNT1 expression. Mechanistically, the interaction site between KLF3 and WNT1 promoter leads to an upregulation in WNT1 transcription, thereby inducing stimulation of the WNT/β-catenin axis; in contrast, when WNT1 is inhibited, it mitigates the proliferative, migratory, and invasive capacity of CRC cells induced by suppressed KLF3 expression. These findings highlight the significance of KLF3 as a novel therapeutic target in CRC and further emphasize the significance of comprehending the regulatory function of transcription factors in the advancement of cancer. The model of the mechanism is described in Figure 8.

KLF transcription factors possess structurally similar zinc finger motifs with specific affinity towards DNA regions rich in guanine-cytosine sequences [4]. KLFs significantly impact various crucial biological processes by regulating gene expression during transcription [24, 25]. A negative prognosis has been linked to elevated levels of KLF6 expression in different forms of cancer, such as in prostate, lung, and ovarian cancer [26–28]. Like other members of the KLF family, KLF3 can function as both a transcriptional activator and repressor through its interactions with other factors [29, 30]. While the involvement of KLF3 in the development of the hematopoietic system has been extensively studied, its role in cancer remains relatively yet to be elucidated. Analysis of clinical samples revealed downregulation of KLF3 in CRC tissues. Patients with low KLF3 expression exhibited worse prognostic outcomes compared to those with high expression. Thus, KLF3 can act as a diagnostic and therapeutic modality for the CRC treatment. For deeper insights into the impact of KLF3 on cancer, we examined how KLF3 influences the proliferation, motility, and invasiveness of the cells and unveiled that reduced KLF3 expression facilitated the manifestation of these malignant characteristics in CRC cells. The aforementioned findings underscore the important contribution of KLF3 in tumor progression. To conduct a more comprehensive investigation into the possible function of KLF3 in CRC, we conducted a Gene Set Enrichment Analysis (GSEA) and examined pertinent literature. The analysis demonstrated the essential function of the WNT/β-catenin axis in driving the process of CRC. The WNT signaling pathway exerts a substantial influence on various physiological processes, such as differentiation, proliferation, and determination of cellular fate [31]. Next, the levels of the markers related to the WNT/β-catenin axis in CRC cells proved the crucial contribution of KLF3 in enhancing the expression of p-GSK3β (Ser9) and β-catenin, thereby promoting the activation of the WNT/β-catenin axis. These observations also highlight the interplay between KLF3 and the WNT/β-catenin axis, and shed light on the regulatory role of KLF3 in CRC progression.

Recent studies have uncovered multiple functions of KLF3 in several types of cancers. In lung cancer, KLF3 is deemed an oncogenic factor, and its silencing suppressed cell growth, immigration and invasive capacity, reflecting its role in stomach cancer [11]. On the other hand, the decreased levels of KLF3 in breast cancer, acute myeloid leukemia, and osteosarcoma, and is thus linked to an inferior prognosis in breast cancer [32], acute myeloid leukemia [33] and osteosarcoma [34]. Our research offers valuable insights into the regulatory role of KLF3 in CRC through its transcriptional activation of the WNT1 gene. The findings of this study are contradictory to research by Wang et al. who revealed that KLF3 knockdown inhibited propagation and malignancy of lung cancer and resulted in cell cycle arrest and apoptosis [35]. Based on the current observations, we propose that KLF3 expression may correlate with tumor outcomes. Higher KLF3 levels in CRC could activate cancer-inhibitory pathways, whereas its lower levels activated cancer-promoting pathways, accelerating tumor progression. Tumor-infiltrating immune cells are critical players in tumorigenesis and progression. The tumor microenvironment (TME) comprises a diverse array of immune cells whose presence correlates with clinical outcomes and response to immunotherapy [36]. Kirberg et al. showed that KLF3 expression complements alternative nuclear factor-κB signaling to promote the maturation of B-cells [37]. They also provided insights into the regulatory role of KLF3 in the eosinophil action on adipocytes, which affects thermogenesis [38]. The findings reveal the potential immunomodulatory effects of KLF3 in CRC, potentially providing protection. Investigating the interplay between KLF3 expression and the TME may provide valuable insights into the therapeutic potential of KLF3 and its immunomodulatory role in CRC. Therefore, further verification is required to the role of determine KLF3 in CRC progression.

## CONCLUSIONS

Our study uncovers the critical function of KLF3 in CRC by activating the WNT/β-catenin axis via modulation of WNT1 expression. This activation promotes tumor growth and the invasive ability of CRC. According to these findings, targeted inhibition of KLF3 could potentially serve as a viable therapeutic approach for individuals diagnosed with CRC.

## MATERIALS AND METHODS

### Cell lines and culture conditions

Cell lines (293T, NCM460, HCT116, HT29, SW620, SW480, and DLD1) were acquired from the Cell Bank of the Chinese Academy of Sciences (Shanghai, China). The cells were cultivated at 37° C and 5% CO_2_.

### Lentiviral production and infection

KLF3 overexpression (oe-KLF3) and downregulation in CRC cells were achieved using lentiviral vectors. The GV367 vector was used for constructing oe-KLF3, while the GV248 shRNA lentiviral vector was used for the KLF3 knockdown construct. Two shRNA sequences, sh-KLF3#1 and sh-KLF3#2, were utilized to inhibit KLF3 expression. Lentiviruses were propagated by transient transfection of 293T cells, and the cells that underwent transduction were subjected to selection using a concentration of 0.6 mg/mL puromycin for a duration of 7 days. After 48 h, the expression of genes was assessed through qPCR and western blotting using total RNA extracted and protein from cells.

### RNA isolation and quantitative real-time PCR (qRT-PCR)

Total RNA was extracted by TRIzol (TAKARA Biotechnology, Japan). cDNA synthesis and qRT-PCR were performed using the PrimeScript RT Master Mix and SYBR Premix Ex Taq II (TAKARA Biotechnology, Japan). The reaction was conducted following the guidelines provided by the manufacturer. The qRT-PCR primer sequences are listed in Supplementary Table 1.

### Western blotting

Cells were detached from the culture medium and lysed with lysis buffer. The BCA Protein Assay Kit (Pierce) was utilized for quantification of total protein following relevant protein quantification. Western blotting was performed by a standard protocol. The proteins were separated by 10% SDS–PAGE and transferred onto polyethylene difluoride superfilm (Merck Millipore, Darmstadt, Germany). The membrane was subsequently blocked using a 5% solution of skimmed milk and incubated overnight with primary antibodies at a temperature of 4° C. Following a series of three washing, the membrane underwent an incubation period of 1 h with a secondary antibody. In the final analysis, the protein signals were detected utilizing the enhanced chemiluminescent technology (Thermo Fisher, 34075, USA). The final results were quantified using the ImageJ software.

### Cell proliferation assays

The Cell Counting Kit-8 (CCK-8) was employed to evaluate the cellular proliferative capacity. First, treated cells (2 × 10^3^ cells/well) were cultivated in 96-well plates according to the prescribed experimental conditions. Cell viability was measured at 24-h intervals. Prior to detection, each well was incubated with 20 μL of CCK-8 reagent. Then, absorbance was determined at 450 nm utilizing a spectrophotometer. Ultimately, the cell proliferation was figured by calculating the average optical density (OD) at 450 nm.

### Determination of 5 -ethynyl-2′-deoxyuridine (EdU)

After transfection and cell counting, cells were plated into the 96-well plate for detection of cell proliferation according to the method. To the medium containing cells, 10 μM EdU solution was added and incubated for 2 h. The attached cells were subjected to fixation using a 4% paraformaldehyde solution. Subsequently, they were washed with PBS and then subjected to an additional wash using 0.5% Triton X-100 solution. Subsequently, the cells were appropriately prepared for staining with DAPI. After three PBS rinses, images were captured using a microscope for the calculation of the proliferation rate.

### Colony formation assay

The cellular proliferation capability associated with KLF3 was evaluated by the colony formation assay. Treated cells (1 x 10^3^cells/well) were placed in a culture dish and cultured in standard conditions for 1-2 weeks. Colonies were treated with 4% paraformaldehyde and stained with 0.1% crystal violet dye. The colonies were quantified using ImageJ software.

### Wound healing assay

After the confluence reached more than 90%, the cell layer was gently abraded using a sterile 200 μL pipette tip, and subsequently the detached cells were thoroughly washed. Then, cells were starved for 24 h in a medium containing 1 mg/mL mitomycin C (Sigma). The microscopic images were obtained for further analysis.

### Transwell assay

The cell motility and invasion capabilities were assessed by Transwell assay (Corning). Cells (3×10^5^ per well) were introduced into the upper layer in 200 μL medium without serum while the lower layer was supplemented with 800 μL of complete medium. Following appropriate incubation, the adherent cells in the lower layer were fixed and stained with the corresponding solution according to the instructions. Cells were counted in five random fields. Finally, cell invasion ability was analyzed.

### Chromatin immunoprecipitation (ChIP) assay and luciferase reporter assay

After reaching approximately 80% confluence, cells were fixed in formaldehyde solution. Cellular chromatin was sonicated into small fragments of chromatin within a certain length range for six times under 10s on /10 s off condition, yielding 100-400 bp fragments. The lysates were pretreated with protein A/G beads, and then incubated for 24h in an incubator with specific antibodies. The isolated DNA was reversely crosslinked overnight at 65° C after a series of elutions. The DNA was employed for semi-quantitative PCR or PCR. Before precipitation, the supernatant was collected for input control. The sequences of primers are listed as in Supplementary Table 1.

The luciferase reporter assay was carried out following the manufacturer’s protocol. KLF3 plasmid or the empty vector was co-transfected into HEK293T cells, followed by another 48-h incubation. The activity of target genes was recorded by the Dual Luciferase Assay System (Promega; E1910). The assay was conducted thrice to ensure consistency in results. Supplementary Table 1 includes a schematic of the proposed KLF3 interaction with the WNT1 promoter region.

### Animal model

BALB/c nude mice were obtained from Hunan Slake Jinda Laboratory Animal Technology Co. and assigned randomly to two groups: sh-NC and sh-KLF3#1. HCT116 cells were transfected with sh-NC or sh-KLF3#1. Tumor size was recorded every five days using the formula: volume = length × width^2^ × 0.5. On day 25 post-inoculation, mice were euthanized, and tumors were excised on reaching 10 mm diameter. The experiments carried out with mice adhered to the guidelines outlined in the institutional agreement after obtaining the approval of the Animal Care and Use Committee of Nanchang University.

### Clinical samples and immunohistochemical staining

A cohort of 105 patients with CRC were selected between February 2017 and December 2020 from Nanchang University Second Affiliated Hospital. CRC tissue and corresponding adjacent normal tissue were obtained through surgical excision. All of the patients include in this study had not received any preoperative chemotherapy or radiotherapy. A portion of each sample was soaked in formaldehyde after freezing in liquid nitrogen for the RNA quantification or preparation of sections. IHC analysis was conducted to assess KLF3 expression in CRC and adjacent normal tissue. Demographic and clinical characteristics were collected and clinical correlation analysis was performed. The present investigation was carried out based on the principles outlined in the Declaration of Helsinki and received approval from the Ethics Committee of the Second Affiliated Hospital of Nanchang University. All participants provided informed written consent.

### Gene set enrichment analysis and JASPAR

The signal pathway enrichment method was followed to sequence genes based on their correlation with KLF3 expression. The variations between the elevated and decreased KLF3 expression groups were revealed by the GSEA 4.3 software. Using KLF3 expression level as the phenotype label and by performing 1000 permutations, potential regulatory mechanisms were investigated to provide evidence for signal pathway regulation. Gene sets with a false discovery rate < 0.05 were identified as significantly enriched. Additionally, the JASPAR database (http://jaspar.genereg.net) was utilized in order to investigate distinct DNA-binding motifs associated with transcription factors and DNA-interacting proteins.

### Statistical analysis

The data were imaged using GraphPad Prism 9.0 software (GraphPad, San Diego, USA). The statistical analyses employed an unpaired Student’s t-test to compare two groups, the differences among multiple samples were evaluated by ANOVA. The Spearman correlation coefficient calculation was utilized to assess the associations among variables. The Log-rank test was employed to conduct survival analysis. Statistical significance was attributed to values with a significance level of p<0.05 across all tests.

### Availability of data and materials

The study’s data can be obtained through TCGA (https://portal.gdc.cancer.gov/), JASPAR (http://jaspar.genereg.net) websites.

## Supplementary Material

Supplementary Figure 1

Supplementary Table 1
