# Supplementary material for: KLF3 promotes colorectal cancer growth by activating WNT1
Source: Aging (Albany NY). 2024 Feb 1;16(3):2475–93. doi: 10.18632/aging.205494 (PMC10911342; doi:10.18632/aging.205494)
Supplement: Supplementary Figure 1 [file aging-16-205494-s001.pdf]

SUPPLEMENTARY FIGURE

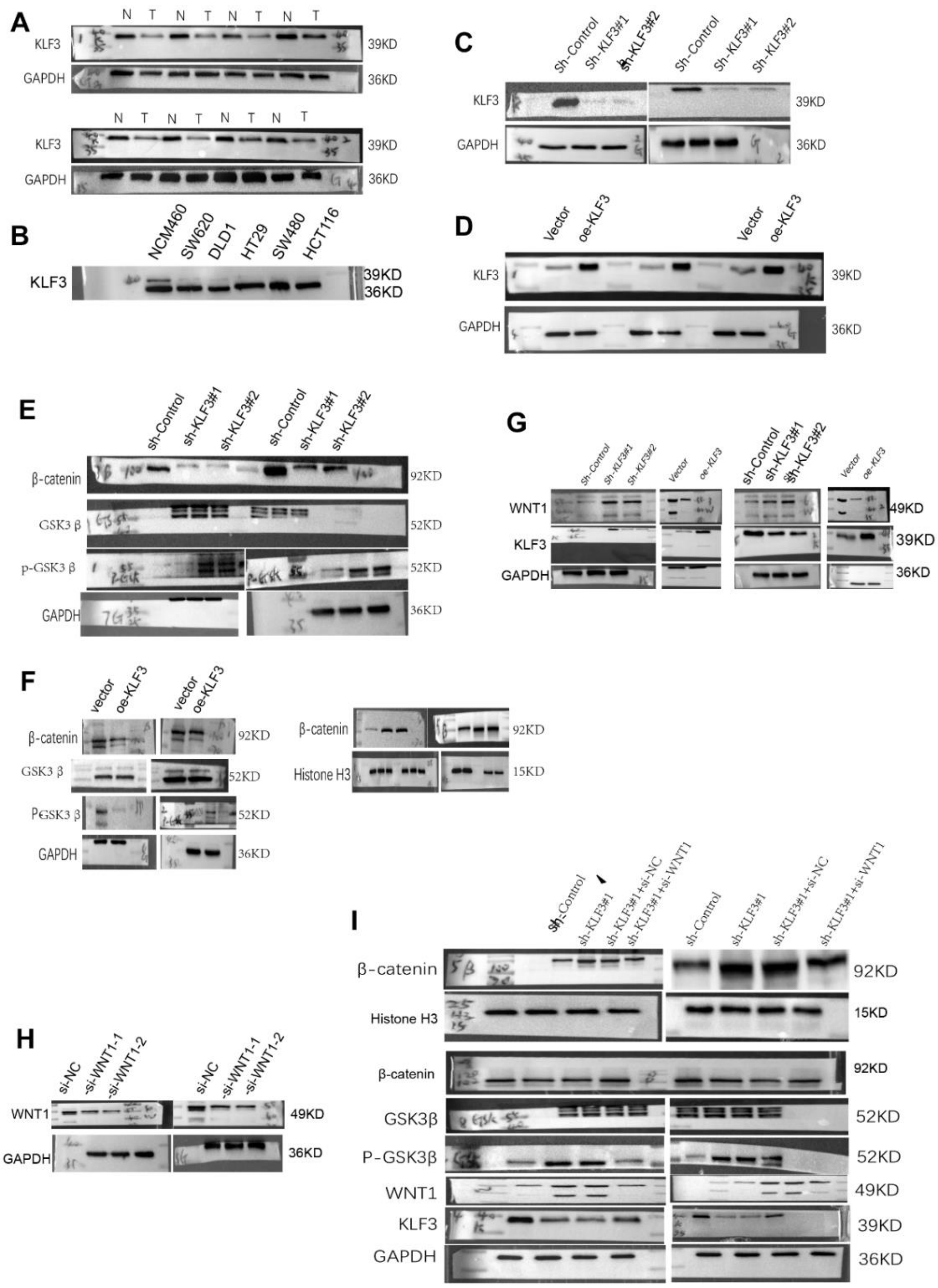

Supplementary Figure 1. The full text refers to the original Western blot image. (**A**) KLF3 protein levels measured by Western blotting in eight pairs of random CRC and normal samples. (**B**) KLF3 expression was higher in NCM460 than five CRC lines. (**C**, **D**) Western blot was used to evaluate the efficacy of knockdown and oe-KLF3 in HCT116 and SW480 CRC cells. (**E**, **F**) In oe-KLF3 and KLF3 knockout CRC cells, protein levels of markers associated with the WNT/β-catenin axis, including GSK3β, p-GSK3β (Ser9) and nuclear β-catenin, were altered. (**G**) Protein levels of WNT1 and KLF3 in HCT-116 and SW480 CRC cell lines after KLF3 knockdown and overexpression, respectively, as shown by Western blotting. (**H**) WNT1 protein levels in CRC cells after transfecting with si-WNT1 or si-NC. (**I**) Levels of markers associated with the WNT/β-catenin axis, including GSK3β, p-GSK3β (Ser9) and β-catenin, were reduced in KLF3 knockdown cells harbouring si-WNT1.
