# Supplementary material for: KLF3 promotes colorectal cancer growth by activating WNT1
Source: Aging (Albany NY). 2024 Feb 1;16(3):2475–93. doi: 10.18632/aging.205494 (PMC10911342; doi:10.18632/aging.205494)
Supplement: Supplementary Table 1 [file aging-16-205494-s002.pdf]

SUPPLEMENTARY TABLES

Supplementary Table 1. Primers, shRNA target sequences and CHIP-qPCR sequences.

Name	Sequences
**Primers for real-time PCR:**	
KLF3 sense	5'-TGTCTCAGTGTCATACCCATCT-3'
KLF3 antisense	5'-CCTTCTGGGGTCTGAAAGAACTT-3'
WNT1 sense	5′-ACGGCGTTTATCTTCGCTATCA-3′
WNT1 antisense	5′-ACGGCCTGCCTCGTTGTT-3′
GAPDH sense:	5'-AGAAGGCTGGGGCTCATTTG-3′
GAPDH antisense:	5'-AGGGGCCATCCACAGTCTTC-3′
The target sites of shRNA:	
sh-KLF3#1 sense	5'-GATCCGATCGAACCACAGAGGACAGATTATTTCAAGAGAATAATCTGTCCTCT GTGGTTCGATCTTTTTTG-3′
sh-KLF3#1 antisense	5'-AATTCAAAAAAGATCGAACCACAGAGGACAGATTATTCTCTTGAAATAA TCTGTCCTCTGTGGTTCGATCG-3′
sh-KLF3#2 sense	5'-GATCCGAGGATACACAGATGTGATTATGATTTCAAGAGAATCATAATCACATC TGTGTATCCTCTTTTTTG-3′
sh-KLF3#2 antisense	5'-AATTCAAAAAAGAGGATACACAGATGTGATTATGATTCTCTTGAAATCATA ATCACATCTGTGTATCCTCG-3′
sh-NC sense	5'-GATCCGTTCTCCGAACGTGTCACGTAATTCAAGAGATTACGTGACA CGTTCGGAGAATTTTTTC-3′
sh-NC antisense	5'-AATTGAAAAAATTCTCCGAACGTGTCACGTAATCTCTTGAATTACGT GACACGTTCGGAGAACG-3′
**Primer name**	**Sequences (5'to3')**
WNT1-Chip1-F	5'-GAGATGGGATTCCACTATGTT-3'
WNT1-Chip1-R	5'-CTTCAAGGCCATTTAGGC-3'
WNT1-Chip2-F	5'-GGGTTCCTGGGCGGTTAA-3'
WNT1-Chip2-R	5'-GCCCGTCGAGGACTAGCAT-3'
